# Assessment of automated pancreatic secretory response measurement in a cohort of pediatric patients

**DOI:** 10.1007/s00261-025-05364-2

**Published:** 2026-01-13

**Authors:** Alexandra O Glenn, Jonathan A Dudley, Maisam Abu-El-Haija, David S Vitale, Andrew T Trout

**Affiliations:** 1 Department of Radiology, Cincinnati Children’s Hospital Medical Center, Cincinnati, USA; 2 Division of Gastroenterology, Hepatology, and Nutrition, Cincinnati Children’s Hospital Medical Center, Cincinnati, USA; 3 Department of Pediatrics, University of Cincinnati College of Medicine, Cincinnati, USA; 4 Department of Radiology, University of Cincinnati College of Medicine, Cincinnati, USA

**Keywords:** Secretin, Pancreatitis, Secretory response, Pancreatic function, Matos criteria

## Abstract

**Objectives:**

Magnetic resonance imaging (MRI) with secretin stimulation allows non-invasive assessment of pancreatic secretion in vivo. Software (PFTquant) can facilitate quantification of secretory response. Our objective was to quantify secretory response for a clinical sample of pediatric patients and compare quantitative to qualitative assessment.

**Methods:**

Two hundred one examinations of 169 patients (< 21 years of age) were processed using PFTquant and manually refined by human observers. Fluid volumes were characterized as normal or abnormal based on 5th percentile values by body surface area (BSA) and age and were compared to qualitative assessment of secretory response.

**Results:**

The mean patient age was 12.1 ± 4.9 (0–20) years and 106 (52.7%) were female. The most common diagnoses were acute recurrent (*n* = 54, 26.9%), chronic (*n* = 53, 26.4%), and acute (*n* = 29, 14.4%) pancreatitis. Based on human-refined segmentation, secretory response was abnormal in 70 (34.8%; by BSA) and 62 (30.8%; by age) patients, including 48.3% (14/29) of patients with acute pancreatitis. Clinical reports characterized secretory response as qualitatively abnormal in 26/201 (12.9%) examinations. Compared to quantitative analysis, qualitative assessment was falsely positive in 12 cases and falsely negative in 50 (BSA) or 55 (age) cases.

**Conclusion:**

In a clinical sample of pediatric and young adult patients, abnormal pancreatic secretory response was present in 30.8 to 34.8% of patients and was most common in patients with acute pancreatitis. Quantiative assessment identifies more patients with abnormal secretory response than qualitative assessment.

## Introduction

Magnetic resonance imaging (MRI) with secretin stimulation of pancreatic fluid secretion allows for non-invasive testing of secretory function [1–3]. Secretory function has been linked to clinical measures of exocrine function including secreted pancreatic fluid volume, pancreatic fluid bicarbonate concentration, and fecal elastase [4–7]. When pancreatic exocrine function is pathologically decreased it can significantly affect growth of children and affect the overall well-being of both children and adults [4, 6, 8–11].

The Matos criteria, involving grading the degree of duodenal filling following administration of secretin, is currently the most widely used method of assessing pancreatic secretory function by MRI but is limitated by subjectivity and lack of evidence for its validity in pediatric patients [1, 2]. Thus, the assessment of pancreatic function by quantification of secreted fluid volumes post administration of secretin may be a more accurate and generalizable test [3–5, 10]. To this end, normal values for secretin-stimulated fluid volumes by MRI have been established for adults with a study of 816 volunteers and for children with a study of 50 healthy children aged 6 to < 16 [10, 11]. The pediatric study defined 5th percentile values by body surface area (BSA) and age to be considered abnormal for children [10]. A single pediatric study has also quantified pancreatic secretory response in a small sample of pediatric patients with suspected pancreatic disease (*n* = 31) [12].

Limitations to adoption of quantitative secretory assessment include the manual effort required to segment images and interrater variability in quantification of secretory response as demonstrated in prior studies [10, 12, 13]. These limitations have been addressed with the introduction of software (PFTquant) that preprocesses and thresholds MR images and performs heuristic detection of non-bowel fluid objects [14]. Users are then able to utilize semi-automated tools in this software to refine the segmentation. Functional performance of this software was demonstrated in a small study in which 20 MRIs performed in children were processed independently by two observers using a manual technique and using PFTquant. PFTquant was found to improve interrater reliability from ICC = 0.69 with manual processing to ICC = 0.90 [14]. Furthermore, time to process was significantly (*p* < 0.001) faster using PFTquant (412 +/- 177 s) compared to manual processing (645 +/- 305 s) [14].

The introduction of software that can decrease effort associated with quantifying secreted fluid volume and improve interrater reliability warrants further analysis in a larger sample. The aims of this study are to (1) apply PFTquant in a large clinical sample, (2) assess the frequencies of abnormal pancreatic secretory response in diagnostic subgroups of patients, (3) compare quantitative analysis of secretin response to qualitative assessment , and (4) characterize the difference in measured fluid volume based on segmentation by the automated software alone versus human-refined segmentation.

## Methods

### Study design and participants

This retrospective study was approved by the institutional review board of Cincinnati Children's Hospital Medical Center (CCHMC) with all study activities conducted in a Health Insurance Portability and Accountability Act (HIPAA) compliant manner. The requirement for documentation of written informed consent was waived.

Clinically performed MRI examinations with secretin were obtained through a search of our clinical picture archiving and communication system (PACS) (Merge PACS; Merative; Ann Arbor, MI). The following inclusion criteria were applied: (1) Examination performed at CCHMC , (2) Examination performed on a Philips MRI machine; (3) Examination performed with secretin administration.

Two hundred and two examinations of 170 patients performed between 12/2019 and 10/2023 were included. One patient that was post-total pancreatectomy with islet autotransplantation was excluded. There is no overlap between the current study cohort and the study cohort used to demonstrate the performance of the PFTquant software [14]. Identified MRI examinations were routed to a secure network storage location for analysis.

### Clinical data collection

Basic demographic information including sex, age at examination, height and weight at examination was extracted from the institutional electronic medical record (EMR) system (Epic; Epic Systems; Madison, WI) and recorded for each patient. Clinical charts were reviewed to determine indication for imaging and any relevant clinical diagnoses at time of imaging. Patients were stratified into the following groups based on their clinical diagnosis as documented in their chart during admission or time of imaging: abdominal pain without pancreatitis; acute pancreatitis; acute recurrent pancreatitis; chronic pancreatitis; exocrine pancreatic insufficiency (EPI) without pancreatitis; biliary/bile duct disorder or disease which included patients with choledocholithiasis, ascending cholangitis, biliary colic, stone disease or any congenital bile duct anomalies such as anomalous pancreaticobiliary junction; primary sclerosing cholangitis (PSC), and an ‘other’ group for patients that did not fit into any of the prior groups.

Clinical MRI reports were reviewed for qualitative assessment of secretory response. In our practice, secretory response is qualitatively assessed by the interpreting radiologist using the criteria defined by Matos et al. for which a grade is assigned dependent on dudodenal filling visualized (0-no fluid, 1-fluid limited to duodenal bulb, 2-fluid partially fills duodenum up to genu, 3-fluid fills beyond genu) and a grade of 0 or 1 is considered abnormal. [1]

### Secreted fluid volume analysis

Bowel fluid volume pre- and post-secretin administration was automatically quantified by PFTquant for each examination with secreted fluid volume automatically calculated as the difference between these volumes [14]. The automated segmentation was then manually refined by a fourth-year medical student (A.O.G.; referred to as R1 going forward) and then further refined by a board-certified pediatric radiologist (A.T.T., 12-years experience; referred to as R2 going forward). Human refinement included removing erroneously included fluid objects or adding missed fluid objects when either were present. Time for each rater to complete their manual corrections was recorded by the software with an internal timer started when the rater initiated a new case and stopped when the case was saved and closed.

### Statistical analysis

Measured secreted fluid volumes for each patient were recorded after automated segmentation, after R1 manually refined the automated segmentation, and after R2 manually refined the segmentation by R1. The final refined fluid volume was defined as the average of the R1 and R2-refined volumes for each patient (R1/R2 Average), assuming ground truth was somewhere in between these two segmentations.

Participant body surface area (BSA) was calculated per Haycock et al. [15] and body mass index (BMI) percentiles were calculated from Centers for Disease Control and Prevention normative data [16] for each patient. Automatically measured secreted fluid volumes and R1/R2 average fluid volumes were used to characterize secretion as normal or abnormal for each patient benchmarked against the published 5th percentile value for BSA and age with frequencies of abnormal values summarized for each patient group [10]. Fifth percentile values were derived from the prior study by Trout et al. that characterized secreted fluid volumes in children aged 6 to < 16 years with BSA values 0.8 to 2.2 m^2^ [10]. For patients in the current study who were < 6 years of age (*n* = 24) or ≥ 16 years of age (*n* = 55) the 5th percentile thresholds for age 6 and 16 years respectively were used. For patients with a BSA < 0.8 m^2^ (*n* = 81) or a BSA > 2.2 m^2^ (*n* = 7) a BSA = 0.8 m^2^ and BSA = 2.2 m^2^ respectively were used.

Interrater agreement for secreted fluid volumes was assessed using intraclass correlation coefficients (ICC) with interpretation as follows: <0.5, poor reliability; 0.5–0.75, moderate reliability, 0.75–0.9 good reliability, and > 0.9 excellent reliability [17]. Bland-Altman difference analyses were performed to quantify the differences in measured fluid volume between raters.

## Results

### Sample characteristics

A total of 201 examinations from 169 patients less than 21 years of age were included. Patient mean age at examination was 12.1 (± 4.9) years. Ninety-two (54.4%) of the patients were female accounting for 106 (52.7%) of the examinations. Mean BMI was 21.6 (± 5.8) kg/m^2^ and mean BSA was 0.98 m^2^ with median BSA = 0.96 m^2^ (range 0.06–2.9). Twenty-four patients were six years of age or younger, all of whom had a BSA < 0.8 m^2^ and 59 other patients over the age of six had a BSA < 0.8m^2^. Fifty-five patients were sixteen years of age or older and 7 patients had a BSA > 2.2 (age range 12–20 years).

**Fig. 1:**
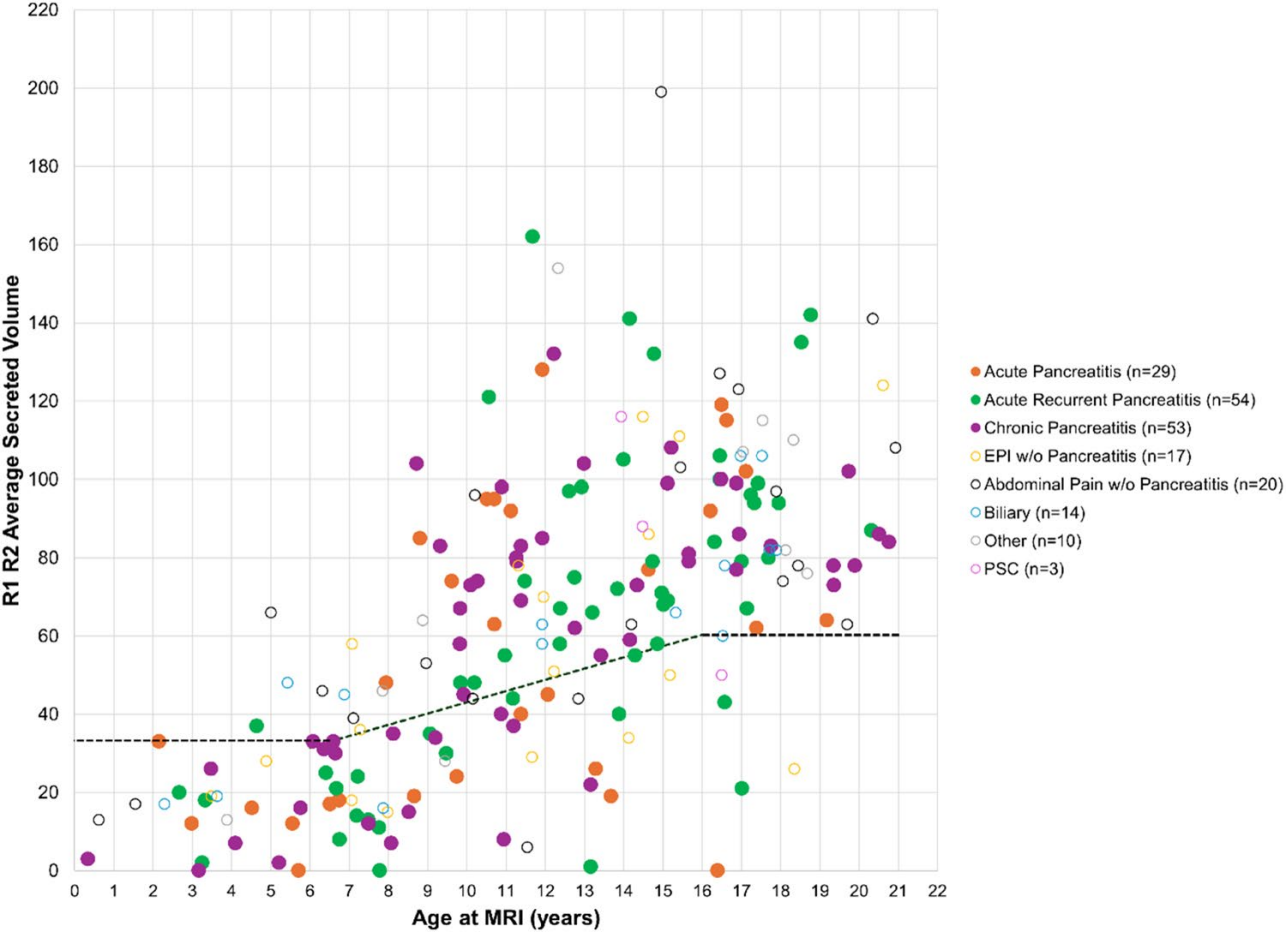
Scatterplot of R1/R2 average measured secreted fluid volumes for patients by age of patient at examination. Diagnosis of patient is delineated. The dashed line indicates the 5th percentile threshold for secretion for age.

**Fig. 2:**
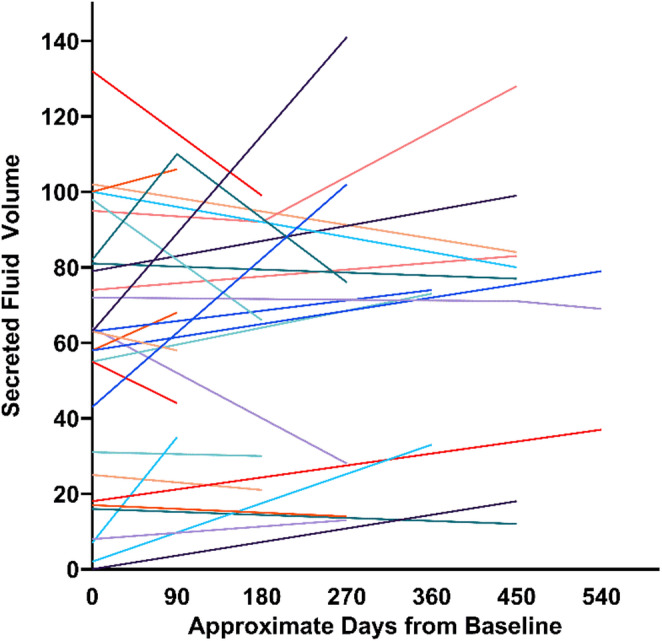
Spaghetti plot of R1/R2 average measured secreted fluid volumes for patients that underwent multiple examinations. In most cases, secreted fluid volumes are relatively flat over time with a few patients showing pronounced increases or decreases

One hundred and thirty-six patients (67.7%) had clinical diagnoses of pancreatitis, 53 (26.4% of 201) of whom had chronic pancreatitis, 54 (26.9% of 201) of whom had acute recurrent pancreatitis, and 29 (14.4% of 201) of whom had acute pancreatitis. Frequencies of other diagnoses are detailed in Table 1. Average secreted volume between R1/R2 by age for patients is shown in Fig. 1. Twenty-nine patients underwent multiple examinations with secreted fluid volumes over time plotted in Fig. 2.

**Table 1:** Baseline characteristics of the study sample (*n* = 169). Values are means and standard deviations or counts and percentages

Variable	Result
Age, years	12.06 (± 4.9)
Female sex, n (%)	106 (52.7%)
BMI percentile*	21.62 (± 5.76)
BSA, m^2^	0.98 (± 0.59)
Diagnosis, n (%) Abdominal pain w/o pancreatitis Acute pancreatitis Acute recurrent pancreatitis Chronic pancreatitis EPI w/o pancreatitis Biliary/Bile duct disease or disorder PSC Other Abdominal pain w/inflammatory bowel disease Abdominal pain with chronic liver & biliary disease Abdominal pain w/maturity-onset diabetes of the young Post pancreatic trauma & pancreatic fluid collection Fatty pancreas Pancreatic transection Post Whipple pancreatitis	21 (10.4%) 29 (14.4%) 54 (26.9%) 53 (26.4%) 17 (8.5%) 14 (7.0%) 3 (1.5%) 10 (5%) 1 (0.5%) 1 (0.5%) 1 (0.5%) 2 (1.0%) 1 (0.5%) 1 (0.5%) 3 (1.5%)
Qualitatively Read as Abnormal Secretin Response	26 (12.9%)

*Three patients were not included in BMI calculations due to being < 2 years of age

### Frequencies of abnormal secretory response

Table 2 displays frequencies of abnormal secretory response based on automatically measured secreted fluid volumes and R1/R2 average secreted fluid volumes, subdivided by patient diagnostic group. Automatically measured secreted fluid volumes were abnormal based on BSA in almost twice as many cases (*n* = 135/201, 67.2%) as R1/R2 average volumes (*n* = 70, 34.8%). More patients had an abnormal secretory response based on BSA-derived thresholds than age-derived thresholds based on automatically measured secreted fluis volumes and R1/R2 average volumes (Table 2).

**Table 2:** Frequencies of abnormal secretory response by observer and judged by BSA or age-defined thresholds for various patient diagnostic subgroups. Results are counts (%) with 95% confidence intervals presented in brackets. R1 refined the automatically generated volumes and R2 further refined R1’s segmentations

	Automatic segmentation		R1-refined volume		R2-refined volume		R1/R2 average volume	
Diagnosis	BSA	Age	BSA	Age	BSA	Age	BSA	Age
Abdominal Pain (*n* = 21)	15 (71.4%) [51.6% − 91.2%]	15 (71.4%) [51.6%−91.2%]	5 (23.8%) [5.1%- 42.5%]	3 (14.3%) [0.0% −29.6%]	5 (23.8%) [5.1%−42.5%]	4 (19.0%) [1.8%−36.3%]	4 (19.0%) [1.8%−36.3%]	3 (14.3%) [0.0%−29.6%]
Acute Pancreatitis (*n* = 29)	22 (75.9%) [60.0%−91.7%]	22 (75.9%) [60.0%−91.7%]	14 (48.3%) [29.8%−66.8%]	13 (44.8%) [26.4%−63.2%]	14 (48.3%) [29.8%−66.8%]	13 (44.8%) [26.4%−63.2%]	14 (48.3%) [29.8%−66.8%]	12 (41.4%) [23.1%−59.6%]
Acute Recurrent Pancreatitis (*n* = 54)	34 (63.0%) [50.0%−76.0%]	34 (63.0%) [50.0%−76.0%]	21 (38.9%) [25.8%−52.0%]	20 (37.0%) [24.0% − 50.0%]	18 (33.3%) [20.6%−46.0%]	17 (31.5%) [19.0%−44.0%]	19 (35.2%) [22.3%−48.0%]	18 (33.3%) [20.6%−46.0%]
Chronic Pancreatitis (*n* = 53)	41 (77.4%) [66.0%−88.7%]	40 (75.5%) [63.8%−87.2%]	18 (34.0%) [21.1%−46.8%]	16 (30.2%) [17.7%−42.7%]	18 (34.0%) [21.1%−46.8%]	15 (28.3%) [16.1%−46.3%]	18 (34.0%) [21.1%−46.8%]	14 (26.4%) [14.4%−38.4%]
Exocrine Pancreatic Insufficiency without Pancreatitis (*n* = 17)	12 (70.6%) [48.3%−92.9%]	12 (70.6%) [48.3%−92.9%]	8 (47.1%) [22.6%−71.5%]	8 (47.1%) [22.6%−71.5%]	8 (47.1%) [22.6%−71.5%]	7 (41.2%) [17.1%−65.3%]	8 (47.1%) [22.6%−71.5%]	8 (47.1%) [22.6%−71.5%]
Biliary Disorder (*n* = 14)	5 (35.7%) [10.6%−60.8%]	4 (28.6%) [8.4%−52.2%]	3 (21.4%) [0.0%−42.9%]	3 (21.4%) [0.0%−42.9%]	4 (28.6%) [8.4%−52.2%]	4 (28.6%) [8.4%−52.2%]	4 (28.6%) [8.4%−52.2%]	4 (28.6%) [8.4%−52.2%]
Primary Sclerosing Cholangitis (*n* = 3)	1 (33.3%) [0.0%−86.7%]	1 (33.3%) [0.0%−86.7%]	1 (33.3%) [0.0%−86.7%]	1 (33.3%) [0.0%−86.7%]	1 (33.3%) [0.0–86.7.0.7%]	1 (33.3%) [0.0%−86.7%]	1 (33.3%) [0.0%−86.7%]	1 (33.3%) [0.0%−86.7%]
Other (*n* = 10)	5 (50.0%) [17.3%−82.7%]	5 (50.0%) [17.3%−82.7%]	3 (30.0%) [0.0%−59.9%]	2 (20.0%) [0.0%−46.1%]	2 (20.0%) [0.0%−46.1%]	2 (20.0%) [0.0%−46.1%]	2 (20.0%) [0.0%−46.1%]	2 (20.0%) [0.0%−46.1%]
**Total** (*n* = 201)	**135 (67.2%)** **[60.7%−73.7%]**	**133 (66.2%)** **[59.6%−72.8%]**	**73 (36.3%)** **[29.7%−43.0%]**	**66 (32.8%)** **[26.3%−39.3%]**	**70 (34.8%)** **[28.2%−41.4%]**	**63 (31.3%)** **[24.9%−37.7%]**	**70 (34.8%)** **[28.2%−41.4%]**	**62 (30.8%)** **[24.4%−37.2%]**

Among patients who had age or BSA outside of the range assessed by Trout et al., 20 of the 24 patients (83.3%) < 6 years of age had abnormal secreted fluid volumes based on the age-derived threshold for a 6 year old and 22/24 (91.7%) had abnormal secreted fluid volumes based on the BSA-derived threshold for a BSA of 0.8 m^2^ using R1/R2 average measured secreted fluid volume. Among the 55 patients who were ≥ 16 years of age, 6/55 (10.9%) had an abnormal measured secreted fluid volume based on the age-derived threshold and 8/55 (14.5%) had an abnormal measured secreted fluid volume based on the BSA-derived threshold.

Among the 81 patients who had a BSA < 0.8 m^2^ (ages 0–13 years), 50/81 (61.7%) had abnormal secreted fluid volumes based on the BSA-derived threshold while 54/81 (66.7%) had abnormal secreted fluid volumes based on age-derived thresholds. Finally, among the 7 patients who had a BSA > 2.2 m^2^, 2/7 (28.6%) had an abnormal measured secreted fluid volume based on the BSA-derived threshold and 0/7 (0%) had an abnormal measured secreted fluid volume based on the age-derived threshold.

### Diagnosis-specific frequencies of abnormal secretory response

Among the 136 patients with pancreatitis, R1/R2 average measured secreted fluid volumes resulted in characterization of secretory response as abnormal in 51 (37.5%) and 44 (32.4%) patients based on BSA and age respectively. Among subtypes of pancreatitis (acute, acute recurrent, chronic) frequencies were similar with overlapping 95% confidence intervals (Table 2).

Among the 17 patients with a clinical diagnosis of exocrine pancreatic insufficiency but without pancreatitis as documented in the patient’s chart by a pediatric gastroenterologist, 47% had an abnormal secretory response by BSA and age based on R1/R2 average volume.

R1/R2 average measured secretory response was classified as abnormal by both BSA and age in 29% (4/14) patients with clinical diagnoses of biliary disorders. Specific diagnoses associated with abnormal secretory responses included: bile salt export pump deficiency, two patients with anomalous pancreatobiliary junction, and biliary stricture.

For the patients with ‘Other’ clinical diagnoses, R1/R2 average measured secreted fluid volume was abnormal in one patient with pancreatic transection and one patient with history of pancreatic trauma.

### Comparison to qualitative clinical assessment

Clinical reports for the included MRI examinations characterized secretory response as qualitatively abnormal in 26/201 (12.9%) examinations. Compared to a reference standard of quantitative analysis based on R1/R2 average measured secreted fluid volume, qualitative assessment failed to identify an abnormal secretory response in 50 examinations based on BSA and 55 examinations based on age (false negatives) and falsely identified an abnormal secretory response in 12 examinations based on both BSA and age (false positives) (Table 3). Accuracy of qualitative assessment relative to the quantitative reference standard was 69% (95%CI: 62.3%−75.5%), sensitivity was 22% (95%CI: 12.5%−34.0%), and specificity 54% (95%CI: 36.4%−70.4%) using the BSA reference. Using the age reference, the accuracy of qualitative assessment was 67% (95%CI: 59.7%−73.1%), sensitivity was 20% (95%CI: 11.6%−31.7%), and specificity was 54% (95%CI: 36.4%−70.4%).

**Table 3:** Confusion matrix comparing qualitative characterization of secretory response via clinical reports for the MRI examinations as compared to a reference standard of quantitative analysis based on R1/R2 average measured secreted fluid volume. Accuracy of qualitative assessment relative to the quantitative BSA-referenced reference standard is 69% (95%CI: 62.3%-75.5%) with 22% (95%CI: 12.5%-34.0%) sensitivity, and 54% (95%CI: 36.4%-70.4%) specificity. Relative to the age-referenced reference standard, the accuracy of qualitative assessment was 67% (95%CI: 59.7%-73.1%) with 20% (95%CI: 11.6%-31.7%) sensitivity and 54% (95%CI: 36.4%-70.4%) specificity

		Quantitative			
		Abnormal by BSA	Normal by BSA	Abnormal by Age	Normal by Age
Qualitative	Abnormal (n=26)	14	12	14	12
	Normal (n=175)	50	125	55	120

### Secreted fluid volume measurement agreement

**Fig. 3:**
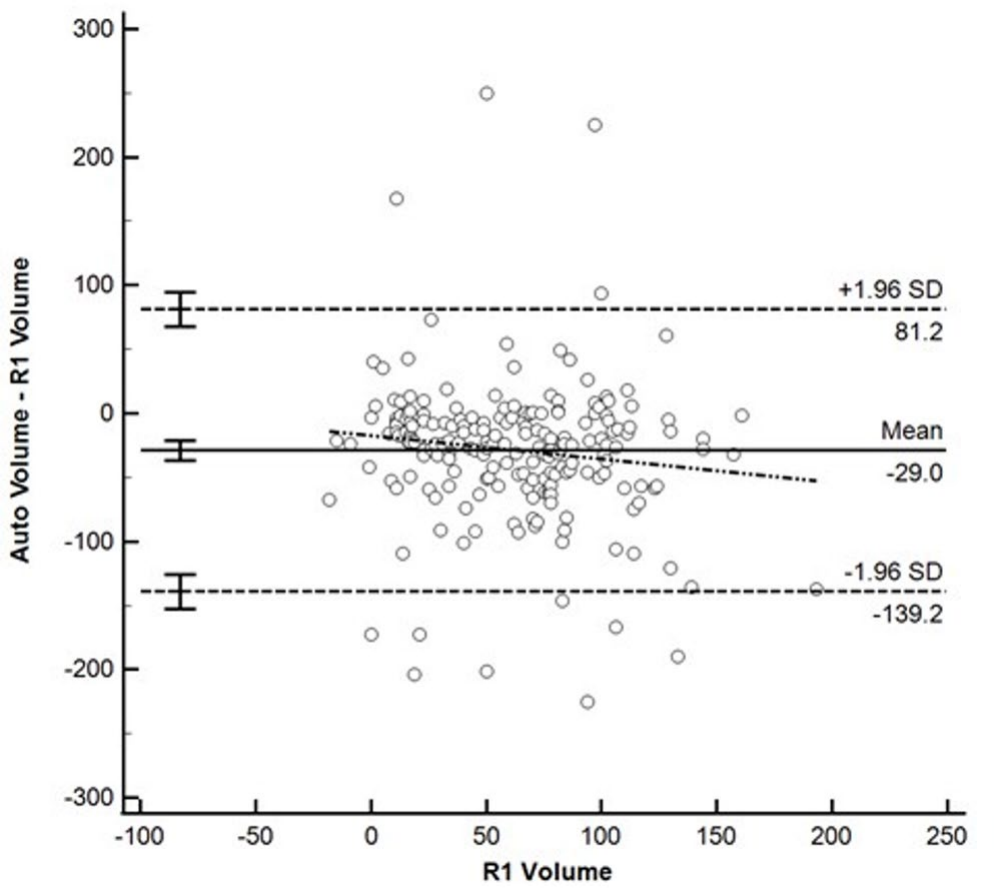
Bland-Altman difference plot for automatically measured secreted fluid volume versus R1-refined secreted fluid volume. Solid horizontal line indicates mean bias with dashed horizontal lines indicating the 95% limits of agreement. Short oblique dashed line reflects simple linear regression of the plotted data and suggests slight negative proportional bias reflecting decreased difference between the automatically measured volume and R1 refined measured volume as the secreted fluid volume increases

**Fig. 4:**
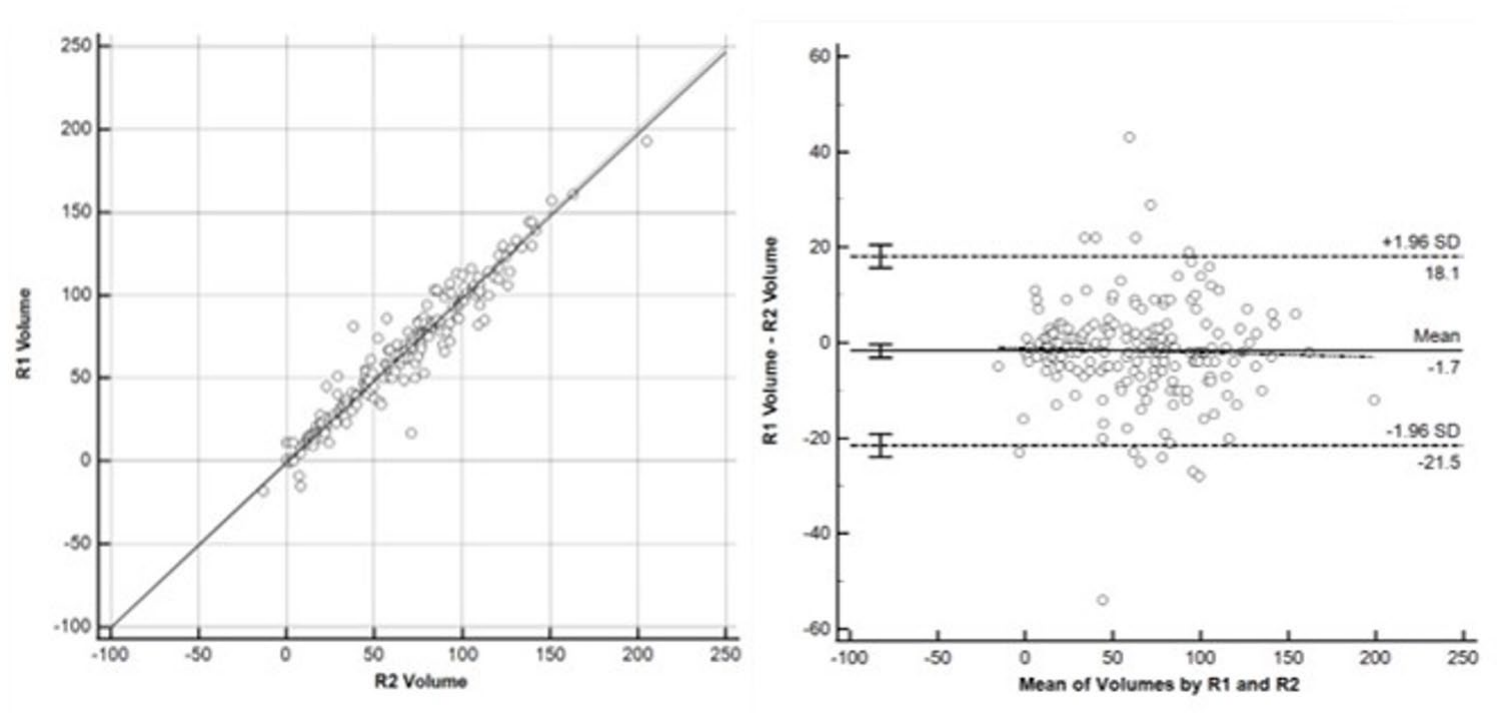
**A** Scatterplot of secreted fluid volumes measured after R1 refinement of the automatic segmentation and R2 refinement of R1’s segmentation. The solid best-fit linear trend line and the dotted line of equality are nearly perfectly superimposed showing little disagreement between observers (ICC=0.96). **B** Bland Altman difference plot for fluid volume measurements based on refinement of the automatic segmentation by R1 and then subsequent refinement of the R1-refined segmentation by R2. Solid horizontal line indicates mean bias with dashed horizontal lines indicating the 95% limits of agreement. Short oblique dashed line reflects simple linear regression of the plotted data and suggests limited proportional bias

**Fig. 5:**
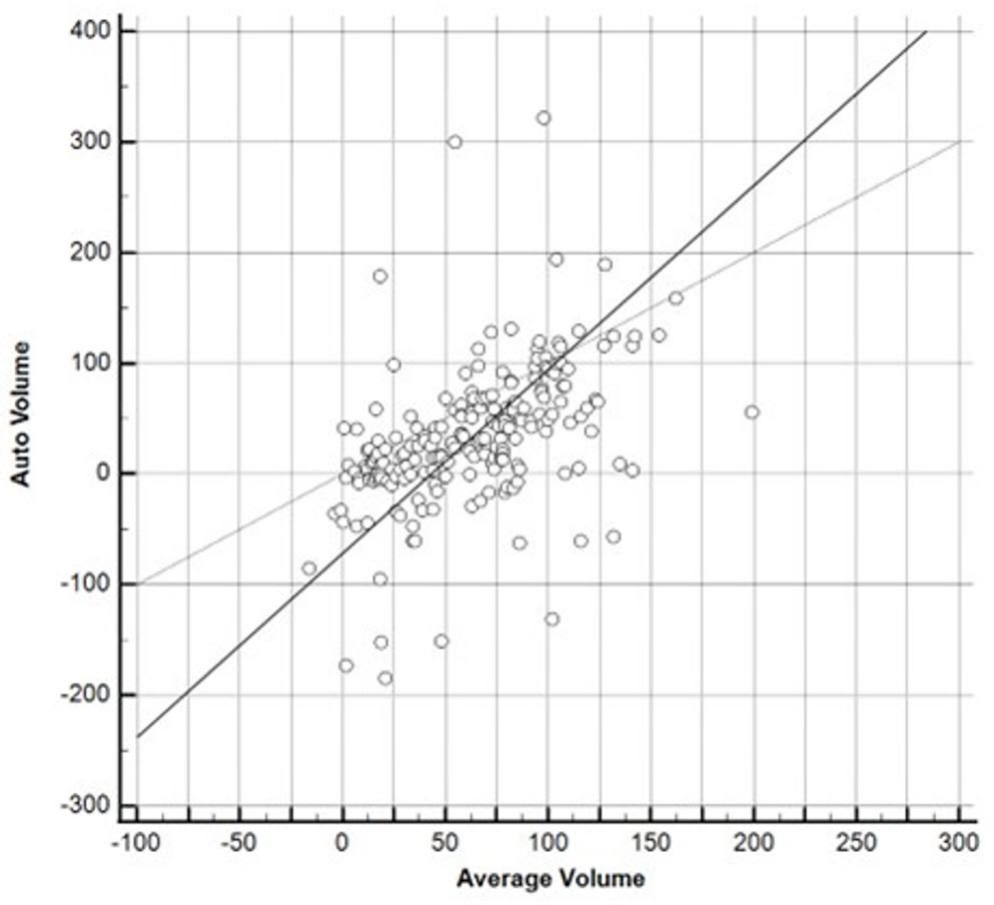
Scatterplot of secreted fluid volumes measured using PFTquant (automatic segmentation) versus the average of volumes after R1 and R2 refinement. Each dot represents a different MRI examination. The solid line is the best-fit linear trend line and the dotted line is the line of equality. In general the automatically measured volume underestimates secreted fluid volume after human refinement (ICC=0.37).

There was poor agreement between measured secreted fluid volume derived from the automatic segmentation and the segmentation refined by R1 (ICC = 0.38; 0.19–0.53 95% CI) with a mean difference in measured volumes of −29.0 mL (95% limits of agreement: −139.2 to 81.2 mL) (Fig. 3), reflecting an average underestimation of secretory response by the automated segmentation. There was excellent agreement between secreted fluid volume derived from the refined segmentation generated by R1 and the further refined segmentation by R2 (ICC = 0.97; 0.95–0.97 95% CI) with a mean difference in measured volumes of −1.7 mL (95% limits of agreement: −21.5 to 18.1) (Fig. 4). Overall, agreement between automatically measured secreted fluid volumes and R1/R2 average volumes was poor (ICC = 0.37; 0.17–0.52 95% CI) (Fig. 5) with a mean difference of − 30.1 mL (95% limits of agreement: (−141.6 to 81.4 mL).

### Processing time

Processing time for R1 was captured for 191/201 data sets with the remainder not captured due to technical difficulties. The average time R1spent refining the automatic segmentation was 5.1 (± 3.2) minutes. Processing time for R2 was captured for 183/201 data sets and the average time spent refining segmentations already refined by R1 was 3.4 (± 1.8) minutes.

## Discussion

Quantification of pancreatic secretory response in response to administration of a secretegogue allows for the non-invasive, objective assessment of pancreatic function by MRI and may have relevance to diagnosing and monitoring pancreatic disease. Based on comparison to normal values for secretory response, it is possible to characterize pancreatic secretory function as normal or abnormal [10]. In this study of 201 MRI examinations clinically performed on 169 pediatric and young adult patients, an abnormal secretory response was present in 34.8% and 30.8% of patients based on BSA-derived or age-derived thresholds respectively. This frequency was largely accounted for by patients with pancreatitis who comprised 80% of our sample and among whom, abnormal secretory responses were identified in 37.5% and 32.4% based on BSA- and age-derived thresholds respecitvely.

In our sample, there was a suggestion of a higher frequency of abnormal secretory response in patients with acute (~ 40–50%) versus acute recurrent (~ 35%) and chronic (~ 25–35%) pancreatitis but these differences were not statistically significant. Among patients with a clinical history of exocrine pancreatic insufficiency without pancreatitis identified either by pancreatic enzyme testing or clinical diagnosis, MRI-quantified secretory response was abnormal in 47.1% of examinations depending on whether age or BSA-derived thresholds were used.

Comparison of quantitative assessment to the commonly clinically implemented qualitative assessment of secretory function by the Matos criteria [1], suggests low sensitivity of qualitative assessment for abnormal secretory response in children and young adults. In our sample, 12.9% (26/201) of the examinations were identified as qualitatively abnormal by the original interpreting radiologist compared to approximately 33% quantitatively identified as abnormal through quantitative analysis. Further, of the 26 examinations with secretory response considered qualitatively abnormal, 12 (46.2%) were false positives and of the 170 examinations with secretory response considered qualitatively normal, 55 (31.4%) were false negatives relative to a reference standard of quantative assessment. This highlights the contribution of quantification of secretory response as a tool to provide a more consistent and sensitive method of measuring secretory response by MRI and reflects the clinical relevance of this technique. With this in mind, we envision that in clinical practice, non-invasive quantitative assessment of secretory response by secretin stimulated MRI, facilitated by semiautomated segmentation, could serve as a screening tool to identify patients who warrant more invasive clincal tests for pancreatic exocrine dysfunction.

Pancreatic secretory response to a secretegogue is thought to reflect pancreatic exocrine function though their remains debate about the strength of association between the two and research into this association is ongoing [4–7, 13]. Pancreatic exocrine function reflects the combination of secteted volume of pancreatic fluid, pancreatic fluid bicarbonate and enzyme concentration and pancreatic enzyme activity. Currently diagnostic techniques for identification of exocrine dysfunction including stool collection and invasive endoscopic sampling are challenged by patient acceptance.

In our sample, automated image segmentation and calculation of secretory response provided a starting point for secretory response quantification but required manual adjustment of the segmentation to avoid underestimation of secretory response and thus false positive results. Human refinement of the automated segmentation resulted in an average change of + 29 mL in calculated secreted volume which decreased the frequency of quantitatively abnormal responses from > 60% to approximately 33%. While the change between the automated segmentation and human refined segmentation was relatively large, the initial automated segmentation allowed the human observer (R1) to produce refined response assessments in an average of 5 min. This was less processing time than was required in the initial description of PFTquant (6.9 min) and was less than half the processing time described for fully manual segmentation in that prior publication (10.8 min), demonstrating the time savings provided by PFTquant [14].

Further demonstrating the contribution of PFTquant, initial human refinement of the PFTquant segmentation in our study was performed by a fourth-year medical student. Additional refinement of the segmentations already refined by R1 by an experienced radiologist produced little change (< 2 mL) on average in the quantified secretory response. This suggests that not only does PFTquant provide time savings when quantitatively analyzing secretory response but it also enables secretory response assessment by relatively inexperienced observers without need for further refinement by experienced observers.

In the current study, we used previously defined age and BSA-derived threshold values to define secretory response as normal or abnormal [10]. Slightly more patients (34.8% vs. 30.8%) were classified as having an abnormal secretory response when using the BSA versus age-derived thresholds. This likely reflects a limitation of the study in that height and weight may not be routinely obtained in clinical encounters and normative data for secretory response have only been defined for a BSA range of 0.8–2.2 m^2^ [10]. In the current study, for patients with BSA outside of this range, we applied fixed threshold values equal to the 5th percentile value for BSA = 0.8 m^2^ for all patients with BSA < 0.8 m^2^ and equal to the 5th percentile value for BSA = 2.2 m^2^ for all patients with BSA > 2.2 m^2^. If secretory responses linearly scale with BSA, this approach may have overestimated the frequency of abnormal secretory response for patients with BSA < 0.8 m^2^ and underestimated the frequency of abnormal secretory response for patients with BSA > 2.2 m^2^.

Our study has further limitations, including the retrospective nature of the study and the inclusion of only MRI examinations acquired on Phillips MRI machinery. Further, there currently exists no clinical reference standard for secretory response or exocrine function applicable to all ages or for specific diagnoses.

In this study of 201 clinically obtained MRI examinations, an abnormal secretory response following secretin administration was quantitatively identified in approximately 1/3 of patients. Comparison of qualitative assessment to quantitative assessment of secretin response found low sensitivity of qualitative assessment by radiologists demonstrating the diagnostic contribution of quantitative analysis to identification of pediatric and young adult patients with impaired secretory response who may have pancreatic exocrine dysfunction. Facilitated segmentation using PFTquant requires human refinement but enables quantitative assessment of secretory response with less human time investment and with excellent agreement between human observers.

## Data Availability

The data that support the findings of this study are not openly available due to reasons of patient privacy and are available from the corresponding author upon reasonable request.
